# A randomized clinical trial of omega-3 fatty acid and vitamin D supplementation on electrocardiographic risk profiles

**DOI:** 10.1038/s41598-023-38344-x

**Published:** 2023-07-15

**Authors:** Jani T. Tikkanen, Elsayed Z. Soliman, Julie Pester, Jacqueline S. Danik, Natalia Gomelskya, Trisha Copeland, I.-Min Lee, Julie E. Buring, JoAnn E. Manson, Nancy R. Cook, Christine M. Albert

**Affiliations:** 1 Research Unit of Internal Medicine, Medical Research Center Oulu, Oulu University Hospital and University of Oulu, Oulu, Finland; 2 Department of Internal Medicine, Epidemiological Cardiology Research Center, Section On Cardiovascular Medicine, Wake Forest School of Medicine, Winston-Salem, NC USA; 3 Department of Cardiology, Smidt Heart Institute, Cedars-Sinai Medical Center, 127 South San Vincente Blvd., AHSP 3100, Los Angeles, CA 90048 USA; 4 Division of Preventive Medicine, Department of Medicine, Brigham and Women’s Hospital, Harvard Medical School, Boston, MA USA; 5 Division of Cardiovascular Medicine, Department of Medicine, Massachusetts General Hospital, Harvard Medical School, Boston, MA USA; 6 Department of Epidemiology, Harvard T.H. Chan School of Public Health, Boston, MA USA

**Keywords:** Nutritional supplements, Epidemiology, Predictive markers

## Abstract

Beneficial and adverse associations with arrhythmias have been reported for omega-3 fatty acids (omega-3 FA) and Vitamin D. The 12 lead electrocardiogram (ECG) contains quantitative measures reflecting diverse aspects of electrophysiology that might provide insights into mechanisms underlying these associations. In a pre-specified ancillary study of the VITaminD and omegA-3 (VITAL) trial, we examined the effect of 1 g of marine omega-3 FA per day, comprised of 460 mg eicosapentanoic acid and 380 mg of docosahexaenoic acid, and 2000 IU VitaminD_3_ per day on ECG characteristics associated with atrial and ventricular arrhythmias among individuals age 50 years or greater. A total of 911 study participants underwent ECGs at baseline and again at 2 years after the randomization. Individuals randomized to active omega-3 FA demonstrated significant net increase in PR-interval duration (*p* = 0.005) and *P*-wave duration (*p* = 0.03) as well significant net decrease in *P*-wave amplitude (*p* = 0.037) as compared to placebo. RMSSD increased to a greater extent in the omega-3 FA arm compared to placebo (*p* = 0.040). For Vitamin D_3_, the Cornell voltage increased to a lesser extent in the participants assigned to active treatment as compared to placebo (*p* = 0.044). There were no other significant differences in QRS, QTc, Cornell voltage or heart rate. Thus, randomized treatment with omega-3 FA supplements resulted in changes on the ECG that are potentially reflective of heightened vagal tone and/or slowing of intraatrial and AV conduction. Vitamin D_3_ supplementation resulted in modest reductions in progressive LV voltage suggestive of a potential antihypertrophic effect.

*Trial registration* ClinicalTrials.gov Identifiers: NCT01169259, NCT02178410 (06/26/2010 and 06/30/2014).

## Introduction

Heart rhythm disorders are major causes of mortality and morbidity globally. Atrial fibrillation (AF) is the most common heart rhythm disturbance and the prevalence is exponentially growing^1^. Sudden cardiac death (SCD) accounts for over 300,000 deaths annually in the US. Experimental^2,3^ and observational studies have suggested that the intake of omega-3 polyunsaturated fatty acids (omega-3 FA) might lower the risk of ventricular arrhythmias and SCD^4,5^ and vitamin D deficiency and/or PTH excess has been associated with increased SCD^6–9^, whereas the observational data regarding atrial arrhythmias are mixed for both.

Three randomized trials of omega-3 FA supplementation have reported protective effects on SCD and ventricular arrhythmias, whereas the remainder have not. With respect to AF, we recently reported that omega-3 FA and/or vitamin D3 supplementation resulted in no significant difference in the risk of incident AF over a median follow up of over 5.3 years in the VITAL trial^10^; however, a small increased risk of AF could not be excluded. In contrast, three recent randomized trials testing various higher dose formulations of omega-3 FA found significantly increased risks of AF in participants randomized to active therapy versus placebo^11–13^. When combined in meta-analysis with 3 additional trials of long term omega-3 FA supplementation, omega-3 FA supplementation was associated with a significant increased risk of AF compared with placebo even at lower dosages of 1 g per day^14^.

The mechanisms underlying this potential increased risk of AF and lower SCD risk associated with omega-3 fatty acid supplementation are unclear, although cellular electrophysiologic and autonomic mechanisms have been postulated^2^. Several parameters on the 12-lead electrocardiogram (ECG) represent potential intermediate phenotypes for heart rhythm disorders, and thus may provide clues regarding potential mechanisms of action^15–17^. Omega-3 FA intake has been favorably associated with several of these parameters in observational studies^18^, however, randomized trial data are lacking. Although Vitamin D supplementation is less well studied with respect to heart rhythm disorders, observational studies have found consistent associations between vitamin D deficiency and clinical conditions predisposing to AF and SCD, most notably including hypertension^19^ and heart failure^20^.

The large-scale randomized placebo-controlled VITamin D and OmegA-3 TriaL (VITAL) primary prevention trial^21,22^ provided a unique opportunity to test the effect of supplementation with omega-3 FA and vitamin D3 on ECG parameters. In a pre-specified sub-study of the VITAL Rhythm trial^10^, serial ECGs were performed at baseline and 2 years after randomization in a subset of 911 participants. Thus, in this present study, we report the effect of both interventions on a variety of pre-specified ECG characteristics previously associated with AF or SCD^17,23^.

## Results

The clinical characteristics and electrocardiographic features of study groups at baseline are presented in Tables 1 and 2, respectively. At baseline, those individuals randomized to active EPA + DHA treatment had significantly higher BMI (*p* = 0.023, Table 1), lower resting heart rate (*p* = 0.033, Table 2), and higher Cornell voltages (*p* = 0.049, Table 2) than those receiving the placebo treatment. Individuals randomized to vitamin D3 treatment had statistically lower diastolic blood pressure (*p* = 0.041, Table 1) and lower mean arterial pressure (*p* = 0.045, Table 1), compared to those in the placebo group. There were no differences in average age, sex distribution, race, smoking status, alcohol intake, or known clinical history of diabetes, hypertension or hyperlipidemia (Table 1) or any of the other ECG parameters (Table 2). Baseline levels of EPA + DHA and vitamin D also did not vary by baseline treatment assignment.

**Table 1:** Clinical characteristics at baseline.

Characteristic	Total CTSC cohort n = 911	EPA/DHA placebo n = 454	EPA/DHA active treatment n = 457	*P* value	Vitamin D3 placebo n = 465	Vitamin D3 active treatment n = 446	*P* value
Age (years), mean ± SD	64.7 ± 6.3	64.7 ± 6.2	64.8 ± 6.4	0.963	65.0 ± 6.4	64.4 ± 6.2	0.158
Male gender, %	52.3	52.0	52.5	0.872	52.7	51.8	0.787
BMI (kg/m2), median (Q1-Q3)	27.4 (24.6–30.6)	27.0 (24.2–30.2)	27.7 (24.8–30.9)	0.023	27.7 (24.7–30.9)	27.2 (24.4–30.1)	0.184
Race (white), %	83.5	84.8	82.3	0.304	83.2	83.9	0.797
Weight (kg), mean ± SD	80.9 ± 17.3	80.0 ± 17.1	81.8 ± 17.4	0.114	80.8 ± 17.4	80.9 ± 17.2	0.912
Waist (cm), mean ± SD	98.0 ± 15.0	97.1 ± 14.7	98.8 ± 15.2	0.078	98.1 ± 15.1	97.9 ± 14.8	0.811
History of diabetes, %	9.8	10.2	9.4	0.705	9.0	10.6	0.437
History of hypertension, %	42.8	40.9	44.6	0.262	45.6	39.9	0.082
History of hyperlipidemia, %	35.4	34.4	36.4	0.524	33.3	37.6	0.184
Smoking (current), %	4.6	5.4	3.9	0.314	4.1	5.2	0.427
Alcohol Intake (≥ 1 drink per day), %	32.7	32.6	32.8	0.962	32.8	32.6	0.954
Glucose (mg/dl), median (Q1–Q3)	93.0 (88.0–101.0)	93.0 (87.0–100.0)	93.0 (88.0–101.0)	0.536	93.0 (88.0–101.0)	93.0 (88.0–99.0)	0.401
Systolic blood pressure (mmHg), mean ± SD	124 ± 14	124 ± 14	124 ± 14	0.668	125 ± 14	123 ± 14	0.094
Diastolic blood pressure (mmHg), mean ± SD	76 ± 9	76 ± 9	77 ± 9	0.306	77 ± 9	76 ± 9	0.041
Pulse pressure (mmHg), mean ± SD	48 ± 11	48 ± 11	47 ± 11	0.164	48 ± 11	48 ± 11	0.636
Mean arterial pressure (mmHg), mean ± SD	100 ± 10	100 ± 11	100 ± 10	0.879	101 ± 10	99 ± 10	0.045
Omega-3 FA blood levels, median (Q1–Q3)	2.70 (2.20–3.50)	2.70 (2.20–3.45)	2.70 (2.30–3.50)	0.464	2.70 (2.30–3.50)	2.70 (2.20–3.40)	0.726
Vitamin D blood levels, median (Q1–Q3)	28.0 (22.0–34.0)	28.00 (23.0–34.0)	28.0 (22.0–34.0)	0.831	29.0 (23.0–34.0)	27.0 (22.0–34.0)	0.097

**Table 2:** Electrocardiographic features at baseline.

Characteristics	Total CTSC cohort	EPA/DHA placebo	EPA/DHA active treatment	*P* value	Vitamin D3 Placebo	Vitamin D3 active treatment	*P* value
Heart rate (bpm), mean ± SD	65 ± 10	66 ± 10	64 ± 10	0.033	65 ± 10	65 ± 10	0.82
PR interval (ms), mean ± SD	172 ± 26	172 ± 25	173 ± 28	0.479	171 ± 25	174 ± 28	0.200
P-wave duration (ms), mean ± SD	114 ± 13	114 ± 12	114 ± 13	0.662	114 ± 13	114 ± 13	0.868
P-terminal force (mV/ms), mean ± SD	2377 ± 2032	2372 ± 1960	2382 ± 2104	0.944	2403.4(2142.8)	2349.0(1911.6)	0.690
P-wave amplitude (mV), mean ± SD	0.123 ± 0.036	0.125 ± 0.036	0.121 ± 0.036	0.084	0.123 ± 0.037	0.123 ± 0.036	0.775
QRS duration (ms), mean ± SD	94 ± 13	93 ± 13	94 ± 14	0.343	94 ± 13	93 ± 13	0.561
QTc interval 1 (ms), median (Q1-Q3)	416 (396–436)	414 (394–436)	420 (396–440)	0.067	417 (396–436)	416 (396–436)	0.877
QTc interval 2 (ms), median (Q1-Q3)	426 (414–438)	426 (412–438)	426 (413–438)	0.706	426 (412–439)	425 (414–438)	0.724
Cornell (mV), mean (Q1-Q3)	1.16 ± 0.54	1.12 ± 0.52	1.19 ± 0.56	0.049	1.18 ± 0.56	1.14 ± 0.52	0.275
SDNN (ms), median (Q1-Q3)	18.6 (12.6–26.8)	19.4 (12.8–27.6)	17.8 (12.6–26.0)	0.237	18.1 (12.6–25.9)	19.3 (12.7–27.7)	0.273
RMSSD (ms), median (Q1-Q3)	18.6 (12.2–27.1)	18.9 (12.2–28.2)	18.3 (12.2–26.3)	0.332	18.1 (12.3–26.2)	19.0 (12.0–28.4)	0.338

QTc intervals, Cornell voltage and SDNN/RMSSD had non-normal distributions.

QTc interval 1 represents QTc calculated with the Framingham formula, QTc interval 2 with the Fredericia formula.

### Effect of EPA + DHA supplementation on ECG measures (Table 3)

**Table 3:** Effect of EPA/DHA treatment on ECG measures.

Normal distribution	Placebo	EPA/DHA		*P*-value
	Mean ± SD	Mean ± SD	Net change % (95% CI)	
PR interval, ms			2.68 (0.82, 4.54)	0.005
Baseline	171 ± 25	173 ± 28		
2-year	173 ± 27	177 ± 29		
*P* wave duration, ms			2.07 (0.20, 3.93)	0.03
Baseline	114 ± 12	114 ± 13		
2-year	108 ± 14	110 ± 15		
*P* wave amplitude, mV			0.004 (0.000, 0.007)	0.037
Baseline	0.125 ± 0.037	0.121 ± 0.036		
2-year	0.121 ± 0.036	0.121 ± 0.036		
*P* terminal force, mVms			64.0 (− 222, 350)	0.66
Baseline	2372 ± 1960	2382 ± 2104		
2-year	3088 ± 2209	3174 ± 2330		
QRS duration, ms			0.21 (− 0.72, 1.14)	0.66
Baseline	93 ± 13	94 ± 14		
2-year	94 ± 13	95 ± 15		
Cornell, mV			− 0.17 (− 0.60, 0.26)	0.44
Baseline	1.12 ± 0.52	1.19 ± 0.56		
2-year	1.25 ± 0.54	1.29 ± 0.55		
Heart rate, bpm			− 0.08 (− 1.02, 0.86)	0.87
Baseline	66 ± 10	64 ± 10		
2-year	64 ± 10	63 ± 10		

Non-normal distribution	Placebo	EPA/DHA		*P*-value
	Median (25–75 percentile)	Median (25–75 percentile)	Net change % (95% CI)	
QTc interval 1, ms			− 0.10 (− 0.73, 0.53)	0.74
Baseline	414 (411–417)	418 (415–420)		
2-year	418 (415–420)	421 (418–424)		
QTc interval 2, ms			− 0.09 (− 0.54, 0.36)	0.68
Baseline	426 (424–427)	426 (424–428)		
2-year	426 (424–428)	426 (424–428)		
SDNN, ms			5.66 (− 2.57, 14.58)	0.18
Baseline	18.7 (17.8–19.7)	18.1 (17.2–19.1)		
2-year	17.6 (16.6–18.6)	17.8 (16.9–18.8)		
RMSSD, ms			8.84 (0.41, 17.97)	0.040
Baseline	18.9 (17.8–20.0)	18.2 (17.2–19.3)		
2-year	18.3 (17.3–19.4)	19.2 (18.1–20.3)		

Treatment effect model adjusted for age, gender, race, BMI, mean blood pressure, and active treatment.

QTc interval 1 represents QTc calculated with the Framingham formula, QTc interval 2 with the Fredericia formula.

Table 3 illustrates the change in analyzed ECG variables within and between treatment groups between baseline and year 2 for those randomized to EPA + DHA and placebo. At year 2, individuals receiving active EPA + DHA treatment demonstrated a significant net increase in PR interval duration. The PR interval increased in both groups, but to a significantly greater extent in those assigned to EPA + DHA (difference in net change, 2.68 ms; 95% CI 0.82 to 4.54; nominal *p* = 0.005). In contrast, the P wave duration decreased in both groups, but to a lesser extent in those randomized to EPA + DHA; resulting in a significant positive net change of 2.07 ms (95% CI, 0.20–3.93; *p* = 0.03) in the comparison between randomized treatment groups. The P-wave amplitude also decreased in those assigned to placebo but remained stable in the EPA + DHA group; resulting in a borderline significant net change between groups (0.004 mV, 95% CI 0.000–0.007). Furthermore, EPA + DHA supplementation was associated with a significant borderline net positive change in the logarithm of RMSSD (net change of 8.84%, 95% CI 0.41–17.97% *p* = 0.040). There was no significant change observed in P terminal force (*p* = 0.66), QRS duration (*p* = 0.66), QTc durations (*p* = 0.74 with Framingham formula and *p* = 0.68 with the Fredericia formula), Cornell voltage (*p* = 0.44), resting heart rate (*p* = 0.87) or SDNN (*p* = 0.18). These results were not materially altered in models additionally controlling for antihypertensive, cholesterol lowering medications, or baseline imbalances in heart rate and Cornell voltage (data not shown).

In secondary stratified analyses, results were similar for all ECG variables among those who reported fish intake above or below the median (1.5 per week). In analyses stratified by baseline EPA + DHA levels, there was nominal evidence for effect modification of the treatment effect on the PR interval. Among those with baseline levels below the median (< 2.7%), the difference in the net change in the PR interval was 4.87 ms (95% CI: 1.97–7.77) in the active versus the placebo group. In comparison, the difference in the net change was only 0.89 ms (95% CI: − 1.52–3.31) in those with EPA + DHA levels > 2.7% (*P*, interaction = 0.039). Tests for interaction were not significant for any of the other ECG variables. In post-hoc analyses, change in EPA + DHA level was significantly associated with change in PR interval in the study population. For each percentage increase in blood level of EPA + DHA, the PR interval increased by 1.55 ms (*p* < 0.001). When the change in EPA + DHA level was added to the multivariable model evaluating the EPA + DHA treatment effect, the covariate for the treatment effect was significantly attenuated and became non-significant; whereas the change in blood level of EPA + DHA remained significantly associated with PR interval (*p* = 0.01).

### Effect of Vitamin D supplementation on ECG measures (Table 4)

**Table 4:** Effect of vitamin D treatment on ECG measures.

Normal distribution	Placebo	Vitamin D		*P*-value
	Mean ± SD	Mean ± SD	Net change (95% CI)	
PR interval, ms			− 0.48 (− 2.35, 1.39)	0.62
Baseline	171 ± 25	174 ± 28		
2-year	174 ± 27	176 ± 30		
*P* wave duration, ms			− 1.49 (− 3.36, 0.38)	0.12
Baseline	114 ± 13	114 ± 13		
2-year	110 ± 14	109 ± 14		
*P* wave amplitude, mV			0.001 (− 0.003, 0.004)	0.67
Baseline	0.123 ± 0.037	0.123 ± 0.036		
2-year	0.121 ± 0.035	0.121 ± 0.037		
*P* terminal force, mVms			− 204.5 (− 490.2, 81.3)	0.16
Baseline	2403 ± 2143	2349 ± 1912		
2-year	3256 ± 2302	3002 ± 2230		
QRS duration, ms			− 0.003 (− 0.93, 0.92)	0.99
Baseline	94 ± 13	93 ± 13		
2-year	95 ± 14	94 ± 14		
Cornell, mV			− 0.44 (− 0.87, − 0.01)	0.044
Baseline	1.18 ± 0.56	1.14 ± 0.52		
2-year	1.31 ± 0.56	1.23 ± 0.53		
Heart rate, bpm			0.44 (− 1.38, 0.50)	0.36
Baseline	65 ± 10	65 ± 10		
2-year	64 ± 10	63 ± 10		

Non-normal distribution	Placebo	Vitamin D		*P*-value
	Median (25–75 percentile)	Median (25–75 percentile)	Net change %(95% CI)	
QTc interval 1, ms			0.42 (− 0.21, 1.05)	0.2
Baseline	416 (413–419)	416 (413–418)		
2-year	419 (416–421)	420 (418–423)		
QTc interval 2, ms			0.15 (− 0.30, 0.61)	0.50
Baseline	426 (424–428)	425 (424–427)		
2-year	426 (424–428)	426 (424–428)		
SDNN, ms			0.08 (− 7. 72, 8.55)	0.98
Baseline	18.0 (17.1–19.0)	18.8 (17.8–19.8)		
2-year	17.3 (16.3–18.2)	18.2 (17.2–19.2)		
RMSSD, ms			0.52 (− 7.28, 8.98)	0.90
Baseline	18.1 (17.1–19.2)	19.0 (17.9–20.1)		
2-year	18.2 (17.2–19.3)	19.3 (18.2–20.5)		

Treatment effect model adjusted for age, gender, race, *BMI* mean blood pressure, and active treatment.

QTc interval 1 represents QTc calculated with the Framingham formula, QTc interval 2 with the Fredericia formula.

Table 4 illustrates the changes in analyzed ECG variables between baseline and year 2 in those randomized to Vitamin D and placebo. There were no statistically significant changes observed between the vitamin D active and placebo treatment groups in any of the investigated ECG measures except for borderline significant difference in Cornell voltage. At year 2, the Cornell voltage increased to a lesser extent in the subjects receiving active vitamin D treatment as compared to those in the placebo-group (*p* = 0.044). Again, additional control for antihypertensive and cholesterol lowering medications did not alter these results. In secondary stratified analyses, these were no significant interactions for vitamin D intake or vitamin D levels for any of the ECG variables.

## Discussion

In this randomized controlled trial, supplementation with omega-3 FA (1 g of EPA + DHA per day) caused several nominally significant changes in ECG parameters over 2-years of follow-up. The PR interval significantly increased and the P-wave duration decreased to a lesser extent in patients randomized to EPA + DHA as compared to placebo. There were also borderline significant net positive differences in change in P-wave amplitude and logarithm of RMSSD in the Omega-3 FA supplementation arm. In the Vitamin D supplementation arm, the Cornell mV, a measure of LVH, increased to a lesser degree than what was observed in the placebo arm. The remainder of the ECG parameters tested did not change with treatment assignment. These observations are novel and provide mechanistic insight on the potential electrophysiologic impact of these commonly used supplements.

Epidemiological data have long provided evidence for an inverse relationship between fish consumption, omega-3 FA, and SCD^24–27^; and there is an extensive body of basic and experimental data suggesting these agents have a multitude of effects on cardiac electrophysiology^2,18^. In cross sectional observational studies and meta-analyses of smaller randomized trials, omega-3 intake and supplementation has been associated with lower heart rate^28^, longer PR interval^18^, and shorter QT interval on ECGs^18,28^, as well as indices of heart rate variability associated with vagal activity^29^. In this randomized trial, we confirmed two of these findings. Omega-3 FA supplementation resulted in prolongation of the PR interval and one measure of heart rate variability, RMSSD; both of which are indicative of an increase in vagal tone. The effects on PR interval and vagal tone have also been observed in experimental studies^30^. These observations are clinically intriguing as measures of heart rate variability and augmented vagal tone have been inversely associated with SCD and VF in experimental and observational studies^31^. However, the relationship between vagal tone and AF is more mixed, with some studies suggesting benefit^32^ while at the same time, subsets of AF are known to be vagally induced. Unlike these prior studies, we did not find any significant effects on heart rate or QTc interval, or any other ECG parameters.

In secondary analyses, we found that the effect of EPA + DHA supplementation on the PR interval was modified by baseline level of EPA + DHA, such that the change in the PR interval was greater in those with EPA + DHA level below the median and the effect was not significant in those above the median, suggesting there may be a threshold effect. Also, we observed that change in EPA + DHA level was strongly associated with change in the PR interval, and when included in the same multivariable model, appeared to mediate the treatment effect consistent with a potential causal association. However, these findings have not been adjusted for multiple comparisons and should be interpreted with caution and considered hypothesis generating.

The PR interval is not only reflective of vagal tone but is also representative of atrial and AV nodal conduction. Omega-3 FA supplementation also had a statistically significant effect on *P*-wave duration, another measure of intra-atrial conduction. These findings suggest that omega-3 FAs might slow intra-atrial conduction, which might in turn increase the vulnerability to AF, offering a potential explanation for the recently reported increase in AF risk in randomized trials testing omega-3 FA supplementation^14^. Increased P-wave duration and PR interval have both been associated with elevated AF risk in observational cohort studies^33^. These data, in combination, underscore the importance of carefully balancing risks and benefits when prescribing fish oil supplementation.

For vitamin D supplementation, we found a marginal protective association between randomized treatment with Vitamin D and progression of LVH on ECG at 2 years, as Cornell voltage increased in both treatment arms, but to a significantly lesser extent in those treated with Vitamin D. These findings are consistent with experimental studies demonstrating direct antihypertrophic actions of the Vitamin D receptor signaling system in cardiomyocytes^34^ and prevention of left ventricular hypertrophy by vitamin D analogs independent of blood pressure in spontaneously hypertensive rats^35^. In a recently published echocardiographic study performed in this same population^36^, a non-significant trend toward less increase in left ventricular mass at two years was observed in the vitamin D as compared to the placebo arms; although development of LVH as defined by LV mass was not specifically examined^36^. With respect to other ECG variables, prior observational studies reported associations between Vitamin D deficiency and prolonged P wave duration, PR interval^37^ and repolarization indices^38,39^; however, we did not observe any clinically significant changes in these ECG variables with active supplementation for vitamin D.

Strengths of this study include the blinded randomized controlled trial design, sample size, a meaningful proportion of African American enrollees, and high rates of adherence with study medications. There are also several limitations to these data. First, it should be acknowledged that this trial tested one specific preparation and dose of omega-3 FA and vitamin D3; and therefore, dose-dependent effects may have gone undetected. Second, we only investigated common, clinically used ECG measures previously associated with arrhythmic events. Thus, it is possible that we might have missed changes in other less standard measures of cardiac conduction, repolarization and/or electrical heterogeneity. Third, follow-up ECGs were only available at one time-point (year 2), limiting the conclusions that can be derived from the data. In particular, we may have missed changes in the ECG reflecting structural alterations that might have required a longer duration of treatment. Fourth, we can’t rule out repeatability issues with the possibility of normal fluctuation of the ECG measures over time^40^. Finally, although the examination of each of the ECG measures was pre-specified for each study agent, we cannot rule out the possibility of a false positive result due to multiple testing.

In conclusion, two years of treatment with omega-3 FA supplementation (1 g EPA/DHA per day) resulted in changes on the ECG that are potentially reflective of heightened vagal tone and/or slowing of intraatrial and AV conduction; whereas, treatment with Vitamin D3 (2000 IU per day) may have exhibited an antihypertrophic effect. These results suggest that these commonly used supplements may have subtle effects on cardiac electrophysiology even at low dosages which may influence, both favorably and adversely, the propensity toward cardiac arrhythmias. Whether such subtle effects could ultimately impact risk for arrhythmias in clinical practice is unknown and requires further study.

## Methods

The VITAL Rhythm study is a substudy of the VITAL trial, a randomized, double-blind, placebo-controlled trial, designed to test effects of vitamin D3 [2000 IU (50 ug)/day] and omega-3 FAs (Omacor^®^ 1 g/day; 460 mg of EPA and 380 mg of DHA) upon incidence of cancer and cardiovascular disease. The trial consented, enrolled, and followed 25,871 randomized participants over a median of 5.3 years^41^. Briefly, nationwide recruitment of men age 50 years and women age 55 years began in March 2011. More than 20% African American enrollment was obtained. In this 2 × 2 factorial trial, 25% of participants were randomized to each arm. The primary results on cardiovascular and cancer endpoints along with the report of the side-effect profile of these agents have been reported previously^21,22^

The VITAL and VITAL Rhythm study are registered at clinicaltrials.gov (NCT01169259; NCT02178410). All participants provided written informed consent. Experimental protocols and methods utilized for both studies were approved by the Institutional Review Board of the Brigham and Women’s Hospital. Before randomization, participants completed a questionnaire assessing demographic, clinical, and lifestyle factors. Participants also completed a modified food frequency questionnaire that assessed dietary, fortified, and supplemental sources of vitamin D and omega-3 FA-intake. Participants agreed to agree to limit consumption of supplemental vitamin D and calcium to no more than 800 IU/d and 1200 mg/d, respectively, from all supplemental sources and to forgo the use of fish-oil supplements during the trial. Blood samples were collected at baseline and at 2-years of follow-up, and plasma phospholipid omega-3 fatty acids (EPA + DHA as a percentage of total plasma phospholipid fatty acids) and serum 25-hydroxyvitamin D assays were measured in stored samples using liquid chromatography-tandem mass spectrometry-mass spectrometry (L-MS/MS) by Quest Diagnostics. The trial protocol has been described elsewhere in detail^41,42^.

The ECG study was performed in a subcohort of 1054 VITAL participants who lived within driving distance of Boston, Massachusetts and participated in detailed in-person health assessments at the Center and Translational Science center (CTSC) at baseline and year 2 of the trial. The CTSC cohort was somewhat younger and healthier than the overall study population, with lower prevalence of obesity, hypertension, diabetes, current smoking, and physical inactivity^41^. The baseline visits took place between January 2012 and March 2014, and the two-year follow-up visits occurred between October 2014 and July 2016. ECGs were obtained on 1034 of 1054 participants (98%) at baseline and 959 out of 964 (99%) at year 2.

During each visit, three digital 10-s consecutive ECGs were obtained using GE MAC 1200 system (GE, Milwaukee, Wisconsin, USA). The first ECG was designated as the main ECG and all electrocardiographic measures were taken from this ECG. The two additional ECGs were used in the determination of two heart rate variability (HRV) measures (SDNN and rMSSD) which were averaged over the 3 ECGs. ECGs were transmitted electronically to the Epidemiological Cardiology Research Center (EPICARE) (Wake Forest School of Medicine, Winston-Salem, NC) for central reading. ECGs were automatically processed, after visual inspection for technical errors and inadequate quality using the 2001 version of the GE Marquette 12-SL program (GE, Milwaukee, Wisconsin, USA). ECG abnormalities were classified using the Minnesota ECG Classification, and the automatically measured durations and amplitudes of each segment of the ECG waveform in each lead were used to calculate the ECG markers of interest.

Participants with poor quality or missing ECG data at baseline and at year 2 or who had atrial fibrillation, atrial flutter, 2nd and 3rd degree AV block, WPW-syndrome or cardiac pacing on ECG were excluded from the analysis. After exclusions, a total of 911 subjects formed the study population for the current investigation.

For the purpose of this study, several ECG indices were prespecified for analyses according to their prior association with either AF or SCD in observational studies^43,44^. The pre-selected measured included R-R interval^45,46^, PR duration^15,43^, *P*-wave indices^44,47^ (axis, amplitude, duration, area, terminal force), QRS duration^43,48,49^, QT interval^43,49,50^, LVH^43,51^, and heart rate variability (HRV)^32,52^. *P* wave duration, *P* wave amplitude and QRS duration measures were defined as maximum values across all 12 leads. QT interval was corrected for heart rate with two formulas: the Framingham linear regression formula [QT + 0.154 (1–60/heart rate), and the Fredericia formula [QT (heart rate/60)^1/3^]. Cornell voltage criteria was selected to represent left ventricular hypertrophy. Two short term time domain HRV indices were selected for analyses: the standard deviation of all filtered RR intervals over the length of the recording (SDNN); and the root mean square of the difference of successive RRs (RMSSD). These measures were obtained from a 10-s ECG that were processed digitally.

We examined the association between randomized treatment with omega-3 FA and ECG parameters over time (repeated measurements at baseline and year 2). Distributions of continuous ECG variables were tested for normality and *P* values were generated using two-sample t-tests, Wilcoxon tests, or chi-squared analysis, depending on the distribution. Natural logarithm transformations were performed to improve normality as appropriate, and effects are presented as net percent changes for these variables.

For each continuous ECG interval, we used a linear mixed-effects repeated measures model with a time by treatment interaction to estimate the net changes over time between the randomized groups. The 95% confidence intervals (CIs) and nominal *P* values for the main intervention effects were estimated for changes in those indices between baseline and 2 years of follow up within each group. Treatment effect models were adjusted for age, sex, race, and covariates not fully balanced by randomization (BMI and blood pressure at baseline). Secondary analyses stratified according to the median of baseline fish intake, EPA + DHA blood levels, vitamin D intake, and Vitamin D blood levels were performed for the respective treatment effect, and a Z-test was used to compare the 2 subgroups as a test for interaction. Post-hoc analyses were also performed that used linear regression to explore associations between change in EPA + DHA levels and change in the ECG variables. Statistical significance was interpreted from 95% CIs excluding zero and 2-tailed *P* values < 0.05. SAS version 9.4 was used for all analyses.

## Data Availability

All data generated or analyzed during this study are included in this published article [and its supplementary information files].
